# Preharvest CPPU Application Improves Litchi Fruit Storability via Modification of Hormone Profiles and Maintenance of Recalcitrant Seed Vigor

**DOI:** 10.3390/foods15193458

**Published:** 2026-09-28

**Authors:** Liyu Fu, Yahui Song, Maoxin He, Sijie Li, Bin Liu, Wenhao Peng, Fang Fang, Zhaoqi Zhang, Xuequn Pang, Xuemei Huang

**Affiliations:** 1 College of Horticulture, South China Agricultural University, Guangzhou 510642, China; lyfu@stu.scau.edu.cn (L.F.);; 2 State Key Laboratory for Conservation and Utilization of Subtropical Agro-Bioresources/Guangdong Provincial Key Laboratory of Postharvest Science of Fruit and Vegetables/Engineering Research Center for Postharvest Technology of Horticultural Crops in South China, South China Agricultural University, Guangzhou 510642, China; 3 Guilin Agricultural Science Research Centre, Guilin 541006, China; 4 School of Horticulture and Food, Guangdong Eco-Engineering Polytechnic, Guangzhou 510520, China; 5 College of Life Sciences, South China Agricultural University, Guangzhou 510642, China

**Keywords:** Litchi fruit, preharvest CPPU treatment, hormone profiles, fruit storability, seed vigor

## Abstract

Litchi (*Litchi chinensis* Sonn.), an important fruit in south China, rapidly loses commercial value within 3–4 days at ambient temperatures due to poor storability. Previous studies have shown that 5 mg L^−1^ CPPU (N-(2-chloro-4-pyridyl)-N′-phenylurea) application can improve postharvest storage of litchi fruit. In this study, we investigated the improved storability of litchi fruit by CPPU application from the perspectives of fruit maturity and seed vigor. Litchi fruit on the tree were treated with CPPU 2 weeks before commercial maturation. Fourteen days after CPPU application, the treated fruit exhibited 61% higher pericarp chlorophyll, 22% thicker pericarp and 76% lower anthocyanin concentrations than untreated controls, which indicates delayed on-tree fruit maturation. CPPU also remodeled the endogenous hormone profiles in the pericarp and seeds, elevating pericarp IAA and DHZR by 47% and 90%, representing active auxin and cytokinin forms, respectively. During the 12 days of ambient storage of the treated preharvest fruit, CPPU-treated fruit showed reduced pericarp browning, disease incidence, and electrolyte leakage, while maintaining a 72% marketable fruit rate against the control’s 25% and higher antioxidant enzyme (APX) activity, indicating an increase in fruit storability when compared to the control. Moreover, CPPU suppressed the upregulation of eight senescence-related genes, including *LcLOX1.5-1*, *LcLOX1.5-2*, and *LcRNase3*. Notably, seed vigor of the litchi fruit decreased with the progress of storage, while the CPPU treatment maintained higher seed vigor compared to the control. These results suggest that the enhanced litchi fruit storability by preharvest CPPU application may be linked to recalcitrant seed vigor maintenance, hormone profile modulation, and pericarp senescence delay.

## 1. Introduction

Litchi (*Litchi chinensis* Sonn.) is one of the most important and distinctive tropical and subtropical fruits in southern China. Litchi fruits are highly appreciated for their bright red pericarp, translucent milky-white aril, and characteristic flavor and aroma [1]. However, the poor storability and rapid postharvest senescence of litchi fruit result in a very short shelf life, largely owing to pericarp browning, aril deterioration, and disease development, which greatly restrict fruit transportation, marketing, and international trade [1,2]. Therefore, improving litchi fruit storability and delaying postharvest senescence are of great importance for extending its commercial value.

Phytohormones play important roles in regulating fruit maturation, senescence, and postharvest storability [3,4]. In general, with the maturation and senescence of fruits, the levels of growth-promoting hormones, such as gibberellins (GAs), auxins (IAA), and cytokinins (CKs), decrease, whereas the levels of senescence-related hormones, such as ethylene (Eth) and abscisic acid (ABA), etc., increase [3,4]. A large number of studies have demonstrated that fruits with lower maturities present higher postharvest storability [5]. Exogenous growth-promoting hormone or plant growth regulator application has been found to maintain postharvest quality and delay fruit senescence [3]. For example, in sweet cherry, preharvest GA application has been reported to maintain fruit firmness, reduce weight loss, and improve postharvest storability [6]. Frankel et al. found that indole acetic acid (IAA) inhibited chlorophyll breakdown and fruit softening as intact mature green pear ripened, while reducing ethylene production [7]. N^6^-benzyladenine (6-BA) application was reported to delay degreening of calamondin (*Citrofortunella microcarpa*) fruit in both light and dark conditions [8]. CPPU (N-(2-chloro-4-pyridyl)-N′-phenylurea), a synthetic plant growth regulator with strong cytokinin activity, has been applied in fruit crops such as kiwifruit and grape to promote fruit growth and modify ripening-related quality traits [9,10]. In litchi, the effect of CPPU application on fruit quality and storability has also been investigated [11]. Stern et al. [12] first found that, compared with the control fruit, preharvest CPPU-treated fruit stored well for 6 weeks at 1 °C, showing reduced pericarp and aril browning, lower decay indexes, and maintaining an acceptable flavor. Fahima et al. [13] further reported that CPPU increased pericarp thickness and weight, enhanced pericarp elasticity, and reduced surface microcracking, thereby decreasing susceptibility to postharvest water loss and pericarp browning. Preharvest CPPU treatment was also found to suppress fruit pigmentation but had no significant effect on fruit size or TSS accumulation in litchi [2,14]. Taken together, these studies indicate that CPPU application modifies multiple physiological characters of litchi fruits, subsequently improving fruit storability and delaying ripening-related changes [2,12,13,14].

Litchi is a special fruit whose edible portion is fleshy aril, while its seed is a typical recalcitrant seed [15]. Recalcitrant seeds generally have high water content, strong metabolic activity, and the ability to germinate soon after dispersal, which is considered an adaptation to warm and humid tropical or subtropical environments [15,16,17,18]. Guo et al. [19] reported that litchi seed maturation occurred earlier than commercial fruit maturity, and that seeds from fruit at lower maturity stages possessed higher vigor than those from fully mature or over-mature fruit. Another study demonstrated that litchi fruit harvested at a lower maturity stage showed better postharvest storability, closely correlating to their higher seed germination capacity and seed vigor, together with higher levels of growth-related hormones. Metabolically active recalcitrant seeds are regarded as “developing seedlings,” which may function as important sites of hormone synthesis [20] and may continue to participate in hormonal regulation during late fruit development and postharvest storage.

Based on the distinctive structure of litchi fruit and our previous findings [20,21] that fruit storability is highly correlated with the vigor of recalcitrant seeds, we hypothesize that CPPU treatment could potentially modulate postharvest senescence and storability of litchi fruit by affecting the vigor of recalcitrant seeds and endogenous hormone homeostasis. Therefore, in this study, preharvest CPPU treatment was applied to systematically investigate its effects on on-tree fruit development, postharvest storage quality, seed germination vigor, endogenous hormone profiles, and senescence in litchi fruit, to explore whether the improvement in litchi fruit storability induced by CPPU is associated with sustained recalcitrant seed vigor and the regulation of hormone homeostasis. The results may also provide a foundation for future comparative investigations into storability regulation of related (sub) tropical fruits with aril and recalcitrant seeds, such as longan (*Dimocarpus longan* Lour), ackee (*Blighia sapida*), rambutan (*Nephelium lappaceum*), mangosteen (*Garcinia mangostana*), and durian (*Durio zibethinus* Murr.).

## 2. Materials and Methods

### 2.1. Plant Materials and Samples

In this study, the Litchi cultivar ‘Jiazai’ (JZ) was selected as the experimental material from a commercial orchard located in the Conghua district of Guangzhou city, China. For the preharvest CPPU treatment, fruit clusters located at different canopy orientations of uniformly vigorous trees were selected and treated at 75 days after anthesis (DAA, 2 weeks before commercial maturity). In total, 30 fruit clusters were assigned to the CPPU treatment group and another 30 clusters to the control group. Fruit clusters were fully immersed in an aqueous solution containing 5 mg L^−1^ of forchlorfenuron (CPPU) and 0.1% (*v*/*v*) Tween 20 for 1 min, while clusters soaked in distilled water with 0.1% (*v*/*v*) Tween 20 served as the control. All treated fruits remained on the trees until commercial maturity, then were manually harvested, packed in polyethylene (PE) bags, and immediately transported to the laboratory.

The above-described CPPU-treated and control litchi fruit were harvested at the fruit maturation stage (14 days after the treatment) and used for the postharvest experiment to investigate fruit storability. Uniform fruits with consistent size, weight, and pericarp color, and free of mechanical damage, disease, and pest infestation were selected for the experiment. All the control and CPPU-treated fruit were respectively disinfected by immersion in 500 mg L^−1^ prochloraz solution for 5 min. Then, all the fruit were air-dried completely at ambient temperature. The treated and control fruit were respectively equally placed in 12 plastic baskets with 50 fruit for each, and covered with 0.015 mm-thick PE film bags for the postharvest storage. Each basket represents an independent biological replicate for statistical analysis.

All the fruit samples were stored at a constant temperature of 25 °C for 12 days. Seed germination assays and pericarp browning observations were performed, and fruit physiological indices were determined on days 0, 4, 8, and 12 of storage. At each sampling time point, seeds, pericarp and aril samples at the equator section of the fruit were collected and immediately snap-frozen in liquid nitrogen, then stored at −80 °C for subsequent biochemical and molecular analyses. For each time point, samples from 6 fruits from one basket were served as a biological replicate.

### 2.2. Browning Index, Disease Index, and Marketable Fruit Rate of Litchi Pericarp

At the four storage time points (0, 4, 8, and 12 days), 10 fruits were randomly selected from each replicate, with a total of 3 biological replicates. The percentage of brown area on the pericarp of each fruit was first estimated through visual inspection. Browning class assessment was conducted as follows: 0 = absence of browning; 1 = slight browning (browning area less than 5%); 2 = moderate browning (browning area less than 25%); 3 = severe browning (browning area ranging from 25% to 50%); 4 = extreme browning (>50%) [21].

Browning index = Σ (browning class × number of fruits in each class)/total number of fruits.

Decay was determined through visual inspection for fungal growth on the fruit surface. Decay incidence was evaluated using the following classes: 0 = no decay; 1 = slight decay (≤5% surface); 2 = moderate decay (5–20% surface); 3 = moderately severe decay (20–50% surface); 4 = severe decay (>50% surface).

Disease index = Σ (disease class × number of fruits in each class)/total number of fruits.

The marketable fruit rate (%) was calculated as the number of fruit free of mold and rot divided by the total number of fruit. All data were collected in triplicate.

### 2.3. Chlorophyll and Anthocyanin Content of Litchi Pericarp

Following the method described by Wang et al. [22], five fruits were randomly selected from each biological replicate. The fruit pericarp was ground into powder with liquid nitrogen, then 0.2 g of powder was added to a 2 mL tube. Subsequently, 1 mL of pre-cooled 80% (*v*/*v*) acetone was rapidly added to each tube. The mixture was vortexed in complete darkness for 2 min using a shaker, followed by static incubation at 4 °C for several hours. After centrifugation (10,000× *g*, 10 min, 4 °C), the supernatant was carefully transferred to fresh centrifuge tubes, and the residual pellet underwent repeated extraction following the same protocol until the extract became colorless (typically requiring overnight processing). All extraction aliquots were pooled for subsequent analysis. All experimental procedures were conducted under light-protected conditions to prevent chlorophyll degradation.

Chlorophyll quantification was performed spectrophotometrically through measurements of optical density (OD) at 663 nm and 645 nm wavelengths in triplicate.

The chlorophyll concentration was calculated using the following empirical formula:

Chloro phyll concentration (mg L^−1^) = 20.2 × OD_645_ + 8.02 × OD_663_

The chlorophyll content per unit mass was determined by:

Chlorophyll content (mg g^−1^) = [Concentration (mg L^−1^) × Total extraction volume (L)]/Sample mass (g)

The anthocyanin content method was performed as described by [23]. First, 1.0 g of the above-described liquid nitrogen grounded pericarp powder was immersed in 5 mL of 0.1 M aqueous HCl. The supernatant was collected after the initial extraction. The residual material underwent two additional extractions using 5 mL of 0.1 M HCl each, continuing until the extract became colorless. The anthocyanin concentration was determined using a pH differential method [24]. Then, 1 mL of extract was diluted in 5 mL of 0.4 M KCl-HCl buffer of pH 1 or in 5 mL of 0.4 M citric acid–Na_2_HPO_4_ buffer of pH 4.5. The absorbance of the dilutions at 510 nm was measured using a spectrophotometer (Shimadzu UV-2450, Kyoto, Japan).

Anthocyanin content (mg g^−1^) = ΔA × L × V_1_ × m/ε × V_2_/M

where L (cuvette thickness) = 1 cm; V_1_ (diluted extract) = 5 mL; V_2_ (total extract) = 15 mL; m (sample mass) = 1.0 g. ε = 29,600 L mol^−1^ cm^−1^ and M = 445 g mol^−1^ (molar mass of free cyanidin aglycone, not cyanidin-3-glucoside); ΔA = A_510_ (pH 1.0) − A_510_ (pH 4.5).

### 2.4. Water Content, Pericarp Thickness, Malondialdehyde (MDA), and Relative Electrolyte Leakage of Litchi Pericarp

The pericarp of litchi fruit was dried at a temperature of 65 °C until a constant weight (dry weight) was reached. The water content (%) = (fresh weight − dry weight)/fresh weight × 100.

Pericarp thickness was measured at the suture line using a digital vernier caliper. The measurement was repeated three times and the average value was calculated.

Relative electrolyte leakage (REL) was measured according to the method of Lin et al. [25]. First, 20 pericarp discs (diameter = 1 cm) were added to 20 mL of distilled water and kept at 25 °C for 30 min. The initial reading (L0) was measured using a conductivity meter (HI-98304, Hanna, Mauritius). The solution was then heated at 100 °C for 20 min. The final reading (Lt) was measured after cooling the boiled solution to room temperature. The REL was calculated using the formula: REL (%) = L0/Lt × 100.

Malondialdehyde (MDA) content was determined using the thiobarbituric acid method [26]. First, 0.1 g of the powdered sample was thoroughly mixed with 1 mL of PBS buffer (pH 7.8), vortexed for 30 s, and then subjected to ice-bath ultrasonic extraction for 20 min. After centrifugation at 10,000× *g* and 4 °C for 10 min, 0.1 mL of the supernatant was mixed with 0.3 mL of 0.6% thiobarbituric acid (TBA) dissolved in 10% trichloroacetic acid (TCA). The mixture was incubated at 95 °C for 30 min, cooled on ice, and centrifuged at 12,000× *g* and 25 °C for 10 min. Subsequently, the supernatant was measured at 532 nm in a 96-well plate, with the nonspecific absorption at 600 nm subtracted. A blank control was prepared by mixing 0.1 mL of PBS buffer with 0.3 mL of 0.6% TBA solution. Results were expressed as nmol g^−1^ FW.

APX activity was assessed in a reaction mixture containing 0.25 mL of enzyme extraction solution, 2.5 mL of 50 mM PBS (pH 7.5) with 0.1 mM EDTA and 0.5 mM ascorbic acid, and 0.25 mL of 2 mM H_2_O_2_. After mixing, the decrease in absorbance at 290 nm was monitored for 3 min [27].

Catalase (CAT) activity was determined according to the method of [27], with minor modifications. One unit (U) of CAT activity was defined as the amount of enzyme required to decompose 1 μmol of H_2_O_2_ per minute per gram of tissue. CAT activity was expressed as U g^−1^ FW.

### 2.5. Total Soluble Solids (TSS) and Ascorbic Acid (AsA) Content in Litchi Fruit

A digital refractometer (PR-32α, ATAGO Co., Ltd., Tokyo, Japa) was employed to determine the total soluble solids (TSS) content in the aril.

Briefly, 0.1 g frozen litchi aril powder was homogenized in 1 mL pre-cooled extraction solution containing 5% (*w*/*v*) trichloroacetic acid (TCA) and 1 mM EDTA-Na_2_. The mixture was vortexed for 1 min and incubated on ice for 10 min, followed by centrifugation at 8000 rpm and 4 °C for 10 min. A volume of 100 μL supernatant was collected and mixed sequentially with 30 μL 0.5 mol L^−1^ EDTA, 50 μL 0.5 mol L^−1^ acetic acid, 100 μL Fast Blue B salt solution and 720 μL double-distilled water. After thorough mixing, the reaction solution was kept static at 25 °C for 20 min. Subsequently, 200 μL of the mixture was transferred onto a 96-well microplate, and the absorbance value was measured at 420 nm using a microplate reader. The AsA concentration was quantified based on an L-ascorbic acid standard curve, and the final results were expressed as mg g^−1^ fresh weight (FW) [26].

### 2.6. Seed Germination

The seeds were collected by manually removing the fruit pericarp and aril tissues, followed by extensive rinsing under running tap water. The cleaned seeds were then sown in moist soil and maintained in dark conditions at 25 °C. Germination was monitored over a 14-day period, with seeds considered germinated when the radicle reached 2 mm in length. The germination rates were assessed by counting all seeds exhibiting primary root emergence in each replicate every 2 days. At each time point, we established 3 biological replicates containing 20 seeds each. The final germination percentage was then calculated based on these counts.

Germination rate (%) = number of germinated seeds/number of test seeds × 100

### 2.7. Hormone Content of Pericarp and Seeds

Phytohormone profiling was performed in both pericarp and seed tissues of CPPU-treated JZ fruits after 14 days of on-tree development using liquid chromatography–electrospray ionization–tandem mass spectrometry (LC-ESI-MS/MS) performed at Wuhan Metware Biotechnology Co., Ltd. (Wuhan, China) [28], according to a previously described method with minor modifications [29]. The tissue was ground into powder with liquid nitrogen and extracted with methanol: water: formic acid (15:4:1, *v*/*v*/*v*) containing isotope-labeled internal standards. The combined extracts were evaporated to dryness under a nitrogen gas stream, reconstituted in 80% (*v*/*v*) methanol, and filtered (polytetrafluoroethylene, 0.22 μm). Chromatographic separation was performed on an ACQUITY UPLC HSS T3 C18 column (2.1 × 100 mm, 1.8 μm) using water and acetonitrile containing 0.04% acetic acid as mobile phases under gradient elution. Then, ABA (abscisic acid), auxin (indole-3-acetic acid), CK (cytokinin) and GA (gibberellin) contents were detected by MetWare based on the ABSciex QTRAP^®^ 6500 LC–MS/MS platform in multiple reaction monitoring (MRM) mode. Metabolites were identified based on their retention times and characteristic precursor/product ion pairs by comparison with authentic standards. Quantification was performed using external calibration curves with internal standard correction, and pooled quality control (QC) samples were used to evaluate analytical reproducibility [30]. We determined phytohormone concentrations and reported them as ng g^−1^ fresh weight (FW) using an external standard calibration. The experiment was designed with three biological replicates, with each replicate consisting of a composite sample from six individual fruits. A heatmap of phytohormone metabolites was produced using the “pheatmap” package in R version 4.1.0.

### 2.8. Gene Expression Analysis by qRT-PCR

Gene expression analysis was carried out using the samples, consistent with the selection for hormone content measurement. Based on the published litchi genome sequence database (https://www.sapindaceae.com/index.php) accessed on 18 September 2025 and our laboratory’s transcriptomic data (NCBI BioProject accession PRJNA1271286), 8 senescence-associated genes were selected for RT-qPCR analysis based on our previous study screening [20]. Gene-specific primers were designed via the Primer3 online tool (https://primer3.org). The primers were selected until their amplification efficiency and melting curve validation reached standard levels. Their sequences are provided in Supplementary Table S1. RT-qPCR was carried out in technical duplicate using the Bio-Rad CFX96 equipment (Bio-Rad, Hercules, CA, USA) in conjunction with the iTaq Universal SYBR Green Master Mix (Bio-Rad). Each reaction was run with three technical replicates per biological replicate. The RT-qPCR data were analyzed using previously established methods [31]. For normalization, we employed the reference gene *LcActin* (LITCHI007623) as an internal control. The 2^–ΔΔCt^ technique was used to calculate the means of gene expression levels [32].

### 2.9. Statistics

Data were organized using Microsoft Excel 2023 (Microsoft Corporation, Redmond, WA, USA). Statistical analyses were performed using SPSS Statistics, version 22.0 (IBM Corp., Armonk, NY, USA). The data are presented as mean values ± the standard error of the mean (SEM) with three biological replicates. For datasets involving two fixed experimental factors, two-way analysis of variance (ANOVA) was performed to evaluate the main effects of CPPU treatment, on-tree development/storage time, and their interaction. Where significant effects were detected, Tukey’s multiple comparison test was carried out for all pairwise comparisons across every combination of treatment and time point. Differences were considered statistically significant at *p* < 0.05.

## 3. Results

### 3.1. Effects of Preharvest CPPU Treatment on Coloration, Pericarp Thickness, and TSS of Litchi Fruit on Tree

On-tree litchi fruit were treated by CPPU solution (5 mg L^−1^) dip 2 weeks before commercial maturation, at the fruit color break stage. At 0 d after the treatment, no obvious differences in aril TSS value, pericarp thickness or color (an important indicator for litchi fruit maturation) were observed between the control and CPPU-treated fruit, whose pericarp was basically green with less than 10% of red pericarp (Figure 1A), correlated to the relatively high chlorophyll content, while low anthocyanin content and red color incidence a* value. From 7 to 14 d, control fruit showed progressive pericarp reddening, accompanied by a marked increase in a* values and anthocyanin content, with a significant drop in chlorophyll content (Figure 1B). In contrast, no obvious reddening was observed for the CPPU-treated fruit, whose pericarp exhibited significantly lower a* values and anthocyanin content (Figure 1D), with significantly higher chlorophyll content at the corresponding time points (Figure 1C). Rather than the pericarp color, CPPU significantly increased pericarp thickness compared with the control at both 7 and 14 DAA (Figure 1E). No significant difference in TSS was observed between the two treatments at 14 d (Figure 1F). Taken together, preharvest CPPU application delayed pericarp reddening and degreening while increasing pericarp thickness, and exhibited no effect on soluble solid accumulation during on-tree fruit maturation, suggesting a potential role in delaying fruit maturation, particularly pericarp maturation.

### 3.2. Effects of Preharvest CPPU Treatment on Endogenous Hormone Profiles in Litchi Pericarp and Seeds on the Tree

Preharvest CPPU application markedly modified the accumulation patterns of hormones, auxins (IAAs), gibberellins (GAs), abscisic acid (ABA), and cytokinins (CKs) in litchi pericarp and seeds after 14 days of on-tree development, as shown by the hormone metabolome heatmap (Figure 2A; detailed data in Table S2). Tissue-specific hormone profiles were observed in the fruit. Active auxin forms, IPA and IAA, were at higher levels in the seeds than in the pericarp, while multiple inactive auxin derivatives were found to accumulate at higher levels in the pericarp than in the seeds, indicating the higher metabolic activity of the seeds than the pericarp. For the GAs, GA3 (active), as well as GA8 and GA 29, inactive derivates of GA1 and GA 20 respectively, were found in seeds but not in pericarp. Active Zeatin and its derivates were mainly found in the seeds, while other active CK forms, such as kinetin (K) and BAP, were mainly found in pericarp. Four representative bioactive hormones, IAA, GA3 dihydrozeatin riboside (DHZR), and ABA, were further selected for quantitative analysis.

IAA content increased in both the pericarp and seed after the treatment. At 14 d, CPPU-treated fruit exhibited significantly higher IAA content in the pericarp and seed compared with the untreated control (Figure 2B). For GA3, an active GA detected in this study, after 14 d, seed GA3 levels increased to higher levels in CPPU-treated fruit than the control fruit (Figure 2C). For the active cytokinin metabolite DHZR, after 14 d, its level increased in the pericarp of both the control and CPPU treatment after 14 d, with significantly higher concentration in the treated pericarp compared with the control. In contrast, seed DHZR levels were significantly lower in both control and CPPU-treated fruit than at 0 d, with no significant difference between the two treatments (Figure 2D). CPPU treatment significantly increased ABA content in the pericarp compared with the control after 14 d, whereas seed ABA content was markedly reduced to comparable levels between the treatments (Figure 2E).

Overall, preharvest CPPU application modified the endogenous hormone profiles in the litchi fruit characterized by elevated IAA and DHZR in the pericarp, as well as increased IAA and GA3 in seeds. These results suggest that CPPU treatment may help maintain a relatively high growth promoting hormone status, which is associated with higher seed vigor and improved postharvest storability in litchi fruit.

### 3.3. Appearance, Browning Index, Disease Index, and Marketable Fruit Rate of Preharvest CPPU-Treated Litchi Fruits During Postharvest Storage

The preharvest CPPU-treated litchi fruit were harvested at 14 days after the treatment, when the fruit were considered commercially mature. The treated and control fruits were stored at an ambient temperature of 25 °C for 12 days (Figure 3A). At the beginning of storage (0 d), the control fruit were fully red with significantly higher a* values and lower L values than the CPPU-treated fruit, which were partially red fruit, as observed during the on-tree development (Figure 1). During storage, pericarp a* and L* values gradually decreased in both groups. However, CPPU-treated fruit consistently maintained lower a* values and higher L* values than the control at the same storage time points. TSS content declined progressively during storage, with no obvious difference between the two groups except at 12 d, when CPPU-treated fruit showed higher TSS than the control (Figure 3D). From 4 d onward, control fruit exhibited a rapid increase in pericarp browning index and disease index, both of which were significantly higher than those of CPPU-treated fruit at 8 and 12 d of storage (Figure 3E,F). By the end of storage, severe pericarp browning, rot, and fungal decay were observed in the control fruit, resulting in a sharp decline in the marketable fruit rate to approximately 25% at 12 d. In contrast, the CPPU treatment maintained a significantly higher marketable fruit rate, maintaining approximately 72% after 12 d of the storage (Figure 3G). Taken together, preharvest CPPU application alleviated postharvest pericarp browning and disease development and slightly slowed the decline in TSS, indicating that the CPPU treatment enhanced the storability of litchi fruit.

### 3.4. Effects of Preharvest CPPU Treatment on Water Content, Pericarp Thickness, Chlorophyll and Anthocyanin Content in Litchi Pericarp During Postharvest Storage

Dynamic changes in pericarp water content, pericarp thickness, and pigment contents were monitored to clarify the physiological basis of the enhanced storability of the preharvest CPPU-treated litchi fruit (Figure 4). At harvest (0 d), no significant differences in pericarp water content were observed between the control and CPPU-treated fruit. However, CPPU-treated fruit showed significantly greater pericarp thickness, higher chlorophyll content, and markedly lower anthocyanin content than the untreated control. Pericarp water content gradually declined in both groups during storage, whereas the CPPU treatment significantly delayed the water loss and maintained significantly higher pericarp water content from 4 to 12 d (Figure 4A). Pericarp thickness also decreased continuously during storage in both groups, while CPPU-treated fruit consistently retained a thicker pericarp than the control at each time point (Figure 4B). Pericarp chlorophyll content decreased gradually during storage, but the CPPU-treated fruit maintained significantly higher chlorophyll levels throughout the storage period (Figure 4C). The anthocyanin content declined rapidly during the storage for each group, while the CPPU-treated fruit consistently showed markedly lower anthocyanin levels than the control (Figure 4D) due to the repressed anthocyanin accumulation on the tree by the treatment (Figure 1D). Taken together, preharvest CPPU application delayed pericarp water loss, maintained pericarp thickness and higher chlorophyll content during storage. These physicochemical changes may be related to the delayed pericarp browning and improved postharvest storability in the CPPU-treated litchi fruit.

### 3.5. Effects of Preharvest CPPU Treatment on MDA Content, APX Activity and Electrolytic Leakage of Litchi Fruit During Postharvest Storage

Cell membrane integrity and antioxidant activity were evaluated based on MDA content, APX activity, CAT activity, AsA content, and electrolyte leakage to clarify the physiological basis by which preharvest CPPU application enhanced the storability of litchi fruit (Figure 5). At harvest (0 d), CPPU-treated fruit showed significantly lower pericarp MDA content than the control, whereas APX activity was slightly lower in CPPU-treated fruit. No obvious difference in electrolyte leakage, CAT activity, or AsA content were observed between the two groups at this stage.

During postharvest storage from 0 to 12 d, MDA content and electrolyte leakage increased continuously in both groups, indicating progressive membrane lipid peroxidation and loss of membrane integrity during pericarp senescence. CPPU treatment significantly suppressed MDA accumulation at all sampling time points and maintained lower electrolyte leakage than the control at 4, 8, and 12 d, with the most pronounced difference observed at 12 d of storage (Figure 5A,B).

APX activity gradually declined during storage in both groups. Although CPPU-treated fruit exhibited significantly lower APX activity than the control at 0 d, CPPU-treated fruit retained significantly higher APX activity at 4, 8, and 12 d during storage (Figure 5C).

CAT activity declined continuously in the control, whereas it was largely maintained in CPPU-treated fruit, resulting in significantly higher CAT activity in CPPU-treated fruit at 4, 8, and 12 d (Figure 5D). Similarly, AsA content decreased progressively in the control, whereas CPPU-treated fruit maintained significantly higher AsA levels at 4, 8, and 12 d (Figure 5E).

Preharvest CPPU application reduced membrane lipid peroxidation, preserved cellular membrane integrity, and maintained both enzymatic and non-enzymatic antioxidant capacity in the pericarp. These physiological changes were associated with delayed pericarp senescence of the CPPU-treated litchi fruit, which is consistent with their improved postharvest storability.

### 3.6. Expression of Senescence-Related Genes in Litchi Pericarp During Postharvest Storage

Eight senescence-associated genes involved in membrane deterioration (*LcLOX1.5-1*, *LcLOX1.5-2*, *LcBSP*), oxidative stress responses (*LcPOD*, *LcPER4*), RNA degradation (*LcRNase3*), and senescence regulation (*LcAPF2)* were screened from our previously published RNA-seq data of litchi pericarp during postharvest storage [33] and confirmed to be pericarp senescence-related genes by Fu et al. [20]. In this study, the expression levels of all eight of these fruit senescence-related genes gradually increased in the control fruit, showing the highest levels at 12 d (Figure 6). However, the increases in expression were slowed in the CPPU-treated fruit, showing significantly lower levels at 12 d (Figure 6). These results further demonstrate that preharvest CPPU treatment delays pericarp senescence during the postharvest storage.

### 3.7. Effects of Preharvest CPPU Treatment on the Germination Capacity of Seeds from Litchi Fruit During Postharvest Storage

Seed germination capacity was evaluated to determine the effect of preharvest CPPU application on seed vigor during 12 days of postharvest storage (Figure 7). At the beginning of storage (0 d), seeds from both the control and CPPU-treated fruit reached 100% germination after 6 days of incubation. However, seeds from CPPU-treated fruit germinated more rapidly, as indicated by significantly higher cumulative germination rates at 2−4 days of incubation compared with the control seeds. As fruit storage progressed from 4 to 12 d, seed vigor in the control fruit gradually declined, as reflected by delayed germination initiation and a marked reduction in final germination percentage. In contrast, preharvest CPPU treatment alleviated the decline in seed vigor during storage. At each fruit storage time point (4, 8, and 12 d), seeds from CPPU-treated fruit consistently showed earlier germination initiation and higher cumulative germination percentages throughout the incubation period than seeds from control fruit. After 12 d of fruit storage, the final germination percentage of control seeds decreased to below 35%, whereas seeds from CPPU-treated fruit maintained a final germination percentage above 50%. Taken together, preharvest CPPU application helped maintain seed germination capacity (seed vigor) of litchi fruit during postharvest storage.

## 4. Discussion

The present study demonstrated that preharvest CPPU treatment improved the postharvest storability of litchi fruit. Overall, this study indicates that preharvest CPPU treatment delays postharvest quality deterioration of litchi fruit not only by delaying the pericarp’s maturity, but also by maintaining the vigor of recalcitrant seeds and enhancing endogenous growth-promoting hormone levels. These findings extend the conventional understanding of CPPU-induced modulation of litchi fruit quality traits and highlight seed vigor as a potential internal factor linked to postharvest storability in litchi fruit.

Cytokinins are known to promote cell division, maintain cellular activity, and delay senescence through the regulation of developmental and metabolic processes [34,35]. CPPU is a synthetic cytokinin-like plant growth regulator that has been widely used to regulate fruit growth, maturation, and quality-related traits in horticultural crops [9,10]. In this study, preharvest CPPU application 2 weeks prior to commercial maturity modified several on-tree litchi fruit traits, including delayed pericarp reddening, chlorophyll degradation and pericarp thickening, while having a marginal effect on TSS accumulation. These modified traits are consistent with several previous studies on preharvest CPPU application in litchi (Figure 1). Here, we also found that CPPU treatment improved postharvest storage performance during ambient storage. CPPU treatment delayed fruit deterioration as represented by reduced pericarp browning and disease incidence, higher marketable fruit rate, slower water loss, etc., compared to the control fruit (Figure 3 and Figure 4). In addition, CPPU treatment delayed fruit senescence, as indicated by lower MDA accumulation and electrolyte leakage, while maintaining higher APX activity (Figure 5), and repressing the increase in senescence-associated gene expression (Figure 6). These results and the findings of previous studies [2,13,14] suggest that preharvest CPPU application consistently improves the postharvest storability of litchi fruit.

Stern et al. [12] ascribed the improved postharvest storability by CPPU to pericarp anatomy modification via increased pericarp thickness. It is noted that the maturity of litchi fruit is usually assessed by fruit degreening and reddening in the litchi industry. The consistent effect of preharvest CPPU application on delayed reddening and degreening implies its efficient positive effect on the delay of fruit maturity. Extensive studies have demonstrated that fruit maturity is an important internal factor governing postharvest fruit storability [36,37,38]. In litchi, we also reported that fruit with relatively lower maturity presented significantly better postharvest storability [20]. Taken together, we propose here that, aside from the improvement in pericarp structure, maturity delay by preharvest CPPU application is an important contribution to the better storability of litchi fruit.

Pericarp browning, water loss, membrane deterioration, and oxidative damage are major factors limiting litchi fruit storability [1,18]. Thus, the improved storage performance of CPPU-treated fruit may be partly attributed to the preservation of pericarp structural integrity, membrane stability, and antioxidant capacity. In the present study, we also found that, compared to the control fruit, the CPPU-treated fruit had higher seed vigor (Figure 7). Litchi seeds are typical recalcitrant seeds that maintain high water content and metabolic activity but rapidly lose viability after being released from the mother tree [13,15]. Our previous study demonstrated that during the maturation of litchi fruit, the seeds from fruit at relatively lower maturity stages possessed higher seed vigor than those from fully mature or over-mature fruit. Previous studies reported that litchi fruit harvested at a lower maturity stage exhibited better postharvest storability [38]. In a recent study, Fu [20] found that fruit with relatively lower maturity also showed higher storability and presented higher seed germination capacity and vigor. In the present study, compared to the control fruit, better postharvest storability was found for the CPPU-treated fruit, in which the seeds maintained higher germination capacity and showed delayed vigor loss during the 12 days of postharvest storage. Taken together, these results suggest that the vigor of litchi’s recalcitrant seeds may act as a potential regulatory factor associated with fruit postharvest storability.

Endogenous hormone balance may provide a mechanistic link between seed vigor and fruit storability. Auxins, gibberellins, cytokinins, and ABA coordinately regulate fruit development, ripening, and senescence, with growth-promoting hormones generally maintaining developmental activity, whereas ABA often participates in ripening and stress-related senescence responses [4,39]. We recently found that litchi fruit harvested at color-breaker stages had higher contents of growth-promoting phytohormones, CK, GA and auxin, and lower expression of eight senescence-related genes, compared to the mature red fruit [20]. Recalcitrant seeds are metabolically active after maturation and can be regarded as developing seedlings, continuously synthesizing hormones during fruit development and maturation [40]. In this study, seeds from CPPU-treated fruit accumulated elevated IAA, IPA, and GA3 alongside reduced ABA levels at 14 days after treatment; these hormonal profiles coincided with sustained seed germination vigor (Figure 2). Meanwhile, CPPU elevated DHZR and IAA concentrations in pericarp tissue, a shift that could potentially contribute to retarded pericarp senescence [2,13]. In addition, GA8 and GA 29 are inactive GA forms directly converted from active GA1 and GA20 respectively [41,42]. The higher GA8 and GA 29 in the CPPU-treated seeds may reflect the transiently high levels of the active GAs by the treatment (Figure 2). Furthermore, many fruits with recalcitrant seeds displayed a decreasing ABA trend during maturation and senescence [20,43,44]. This trend may be ascribed to the recalcitrant features of recalcitrant seeds, which maintain high seed vigor and are ready to germinate once detached from trees after maturation. The decreasing ABA levels may further promote germination.

CPPU triggered tissue-specific remodeling of endogenous hormone pools in pericarp and seed tissue, which may be correlated with sustained seed vigor (Figure 7) and delayed pericarp senescence. Delayed senescence was evidenced by mitigated loss of cell membrane integrity (Figure 5) and suppressed transcriptional induction of eight senescence-associated genes (Figure 6). On the basis of these correlative datasets, we propose that preharvest CPPU treatment sustains vigor of litchi recalcitrant seeds, which may trigger tissue-specific shifts in endogenous hormone profiles within surrounding fruit tissues (including the pericarp); these coordinated hormonal and physiological changes are associated with improved litchi fruit storability. Furthermore, the present study showed that the preharvest CPPU treatment 2 weeks prior to commercial maturity was an applicable feasible low-cost strategy for improving litchi storage performance. However, regulatory status in export markets, residue considerations, etc., must be considered for further commercial adoption.

## 5. Conclusions

Preharvest CPPU treatment significantly delayed the maturation of litchi fruit, as represented by delayed pericarp pigmentation and enhanced growth-promoting hormone levels. The treatment effectively improved postharvest storability of litchi fruit under ambient storage. Notably, the CPPU-treated fruit retained higher seed vigor throughout postharvest storage. This physiological trait correlated with tissue-specific reprogramming of endogenous hormone profiles. Seeds from CPPU-treated fruit accumulated higher IAA and GA3 alongside lower ABA, while pericarp tissues exhibited elevated DHZR and IAA levels. This study highlights seed vigor as a potential internal factor associated with postharvest storability and provides an applicable preharvest regulation strategy for extending the storage life of litchi fruit.

## Figures and Tables

**Figure 1:**
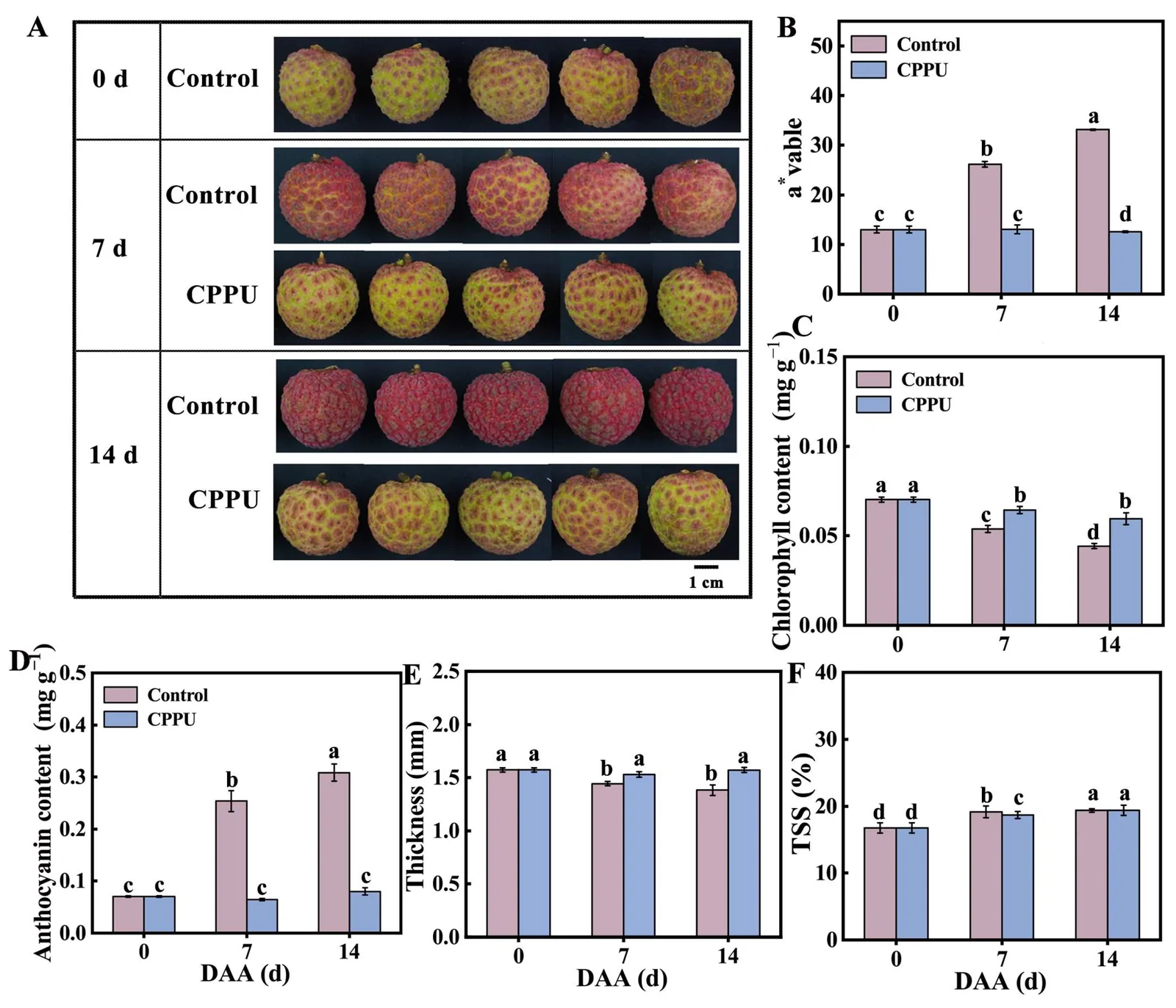
Effects of preharvest CPPU application on fruit appearance and physicochemical attributes of on-tree litchi fruit after treatment (0, 7, and 14 days after application, DAA). (**A**) Representative photographs of litchi fruit under control and CPPU treatments. (**B**–**F**) Changes in pericarp a* value, pericarp chlorophyll content, pericarp anthocyanin content, pericarp thickness, and aril total soluble solids (TSS, %), respectively. Each value represents the means ± SE of three replicates (SE). Different lowercase letters label significant differences at *p* < 0.05, determined by two-way ANOVA followed by Tukey’s multiple comparison test for all combinations of treatments (control and CPPU) and sampling time points (0, 7, and 14 d).

**Figure 2:**
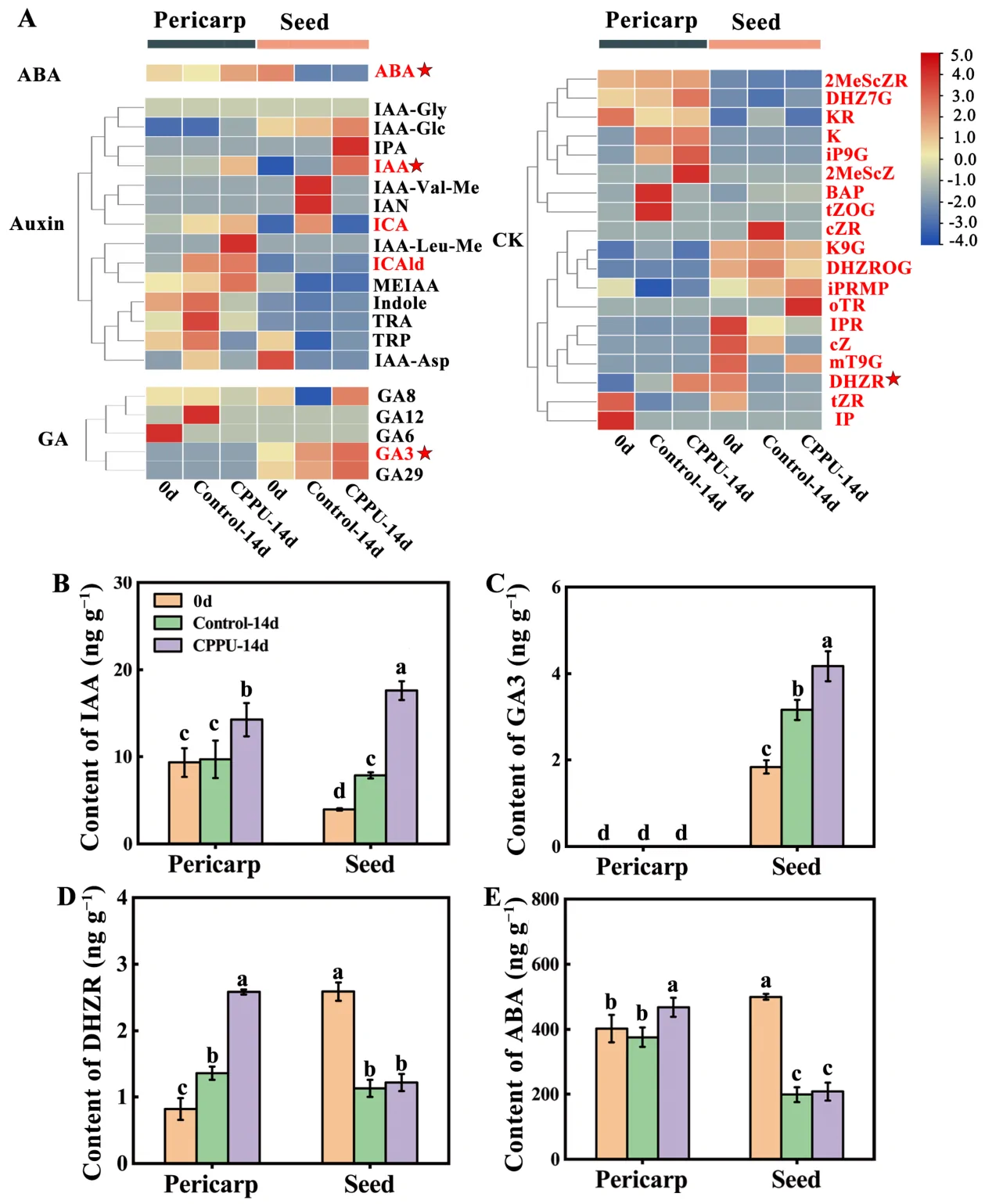
Effects of preharvest CPPU treatment on endogenous phytohormone profiles and major hormone contents in litchi pericarp and seed. Samples were collected at 0 d and 14 days after CPPU application (14 d). (**A**) Heatmap showing the relative accumulation of auxin, gibberellin (GA), abscisic acid (ABA), and cytokinin-related metabolites in the pericarp and seed of the control and CPPU-treated fruit. Compounds marked with asterisks, including IAA, GA3, dihydrozeatin riboside (DHZR), and ABA, are further quantified in panels (**B**–**E**). (**B**–**E**) Quantitative changes in ABA, IAA, GA3, and DHZR contents in pericarp and seed tissues, respectively. The color indicates the accumulation level of each metabolite, from low (blue) to high (red). The hormones marked in red are bioactive, with those marked by asterisks (★) indicating higher concentration compounds. Each value represents the means ± SE of three replicates (SE). Different lowercase letters indicate significant differences at *p* < 0.05, determined by two-way ANOVA followed by Tukey’s multiple comparison test to compare all combinations of tissues (pericarp vs. seed) and treatments (0 d; control—14 d; CPPU—14 d).

**Figure 3:**
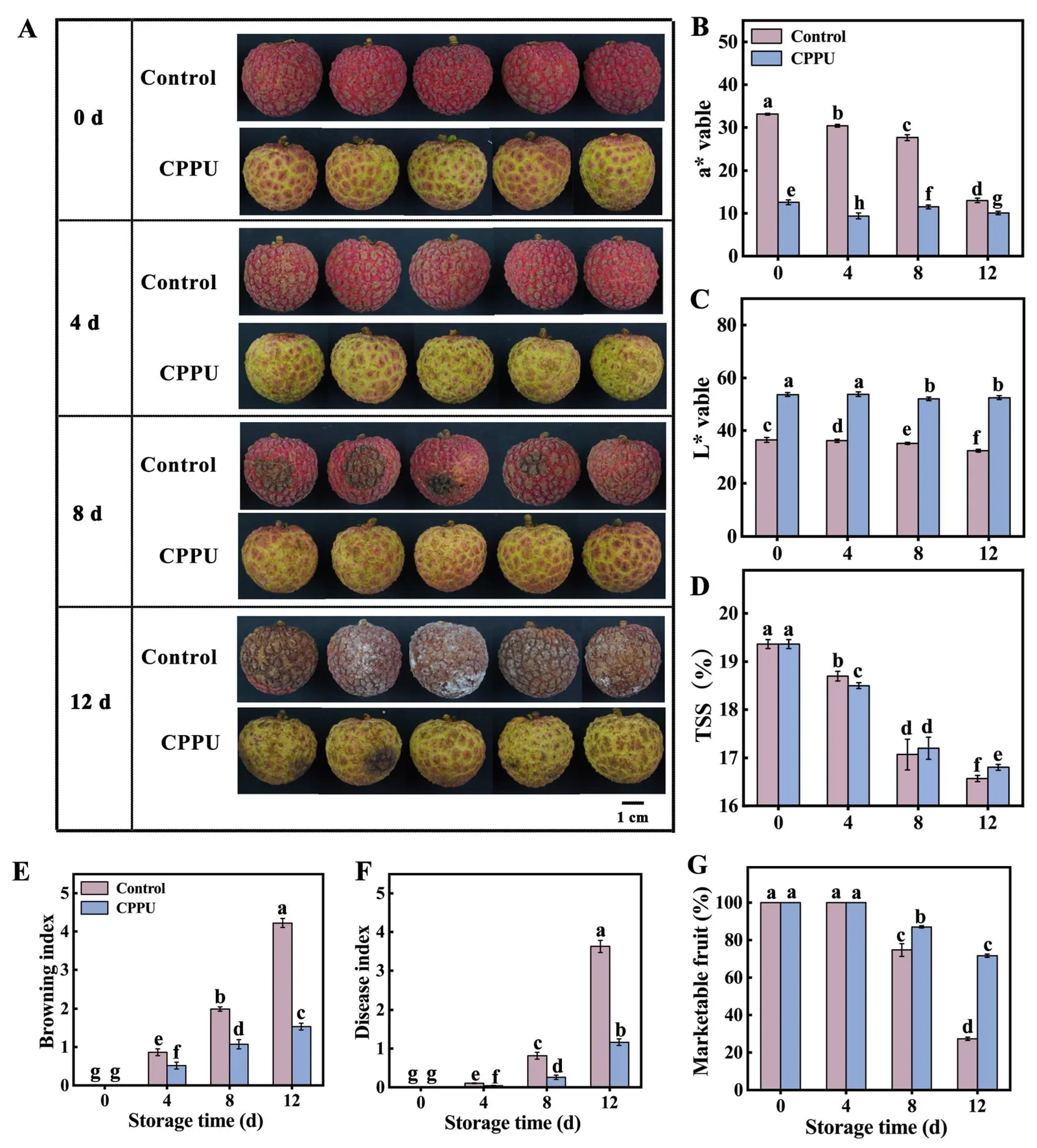
Postharvest storage performance of preharvest CPPU-treated litchi fruit during 12 days of ambient storage. (**A**) Representative fruit appearance of control and CPPU-treated fruit after 0, 4, 8, and 12 d of storage. (**B**–**G**) Changes in pericarp a* value, pericarp L* value, aril TSS, pericarp browning index, disease index, and marketable fruit rate, respectively. Each value represents the means ± SE of three replicates (SE). Different lowercase letters label significant differences at *p* < 0.05, determined by two-way ANOVA followed by Tukey’s multiple comparison test for all combinations of treatments (control and CPPU) and storage time points (0, 4, 8, and 12 d).

**Figure 4:**
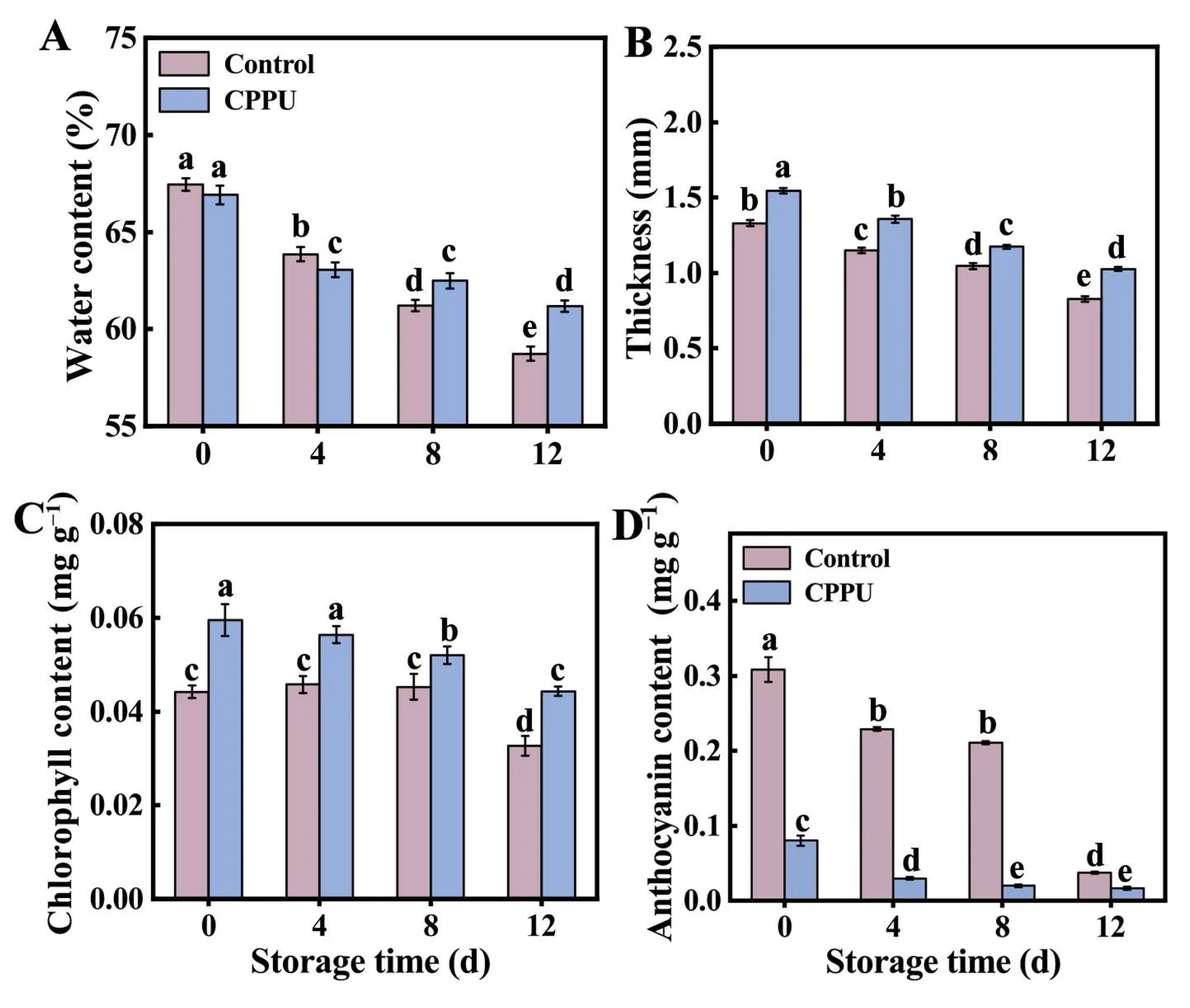
Changes in pericarp physicochemical characteristics of the preharvest CPPU-treated litchi fruit during 12 days of ambient postharvest storage. (**A**) Pericarp water content (%); (**B**) pericarp thickness; (**C**) chlorophyll content; and (**D**) anthocyanin content. Each value represents the means ± SE of three replicates (SE). Different lowercase letters label significant differences at *p* < 0.05, determined by two-way ANOVA followed by Tukey’s multiple comparison test for all combinations of treatments (control and CPPU) and storage time points (0, 4, 8, and 12 d).

**Figure 5:**
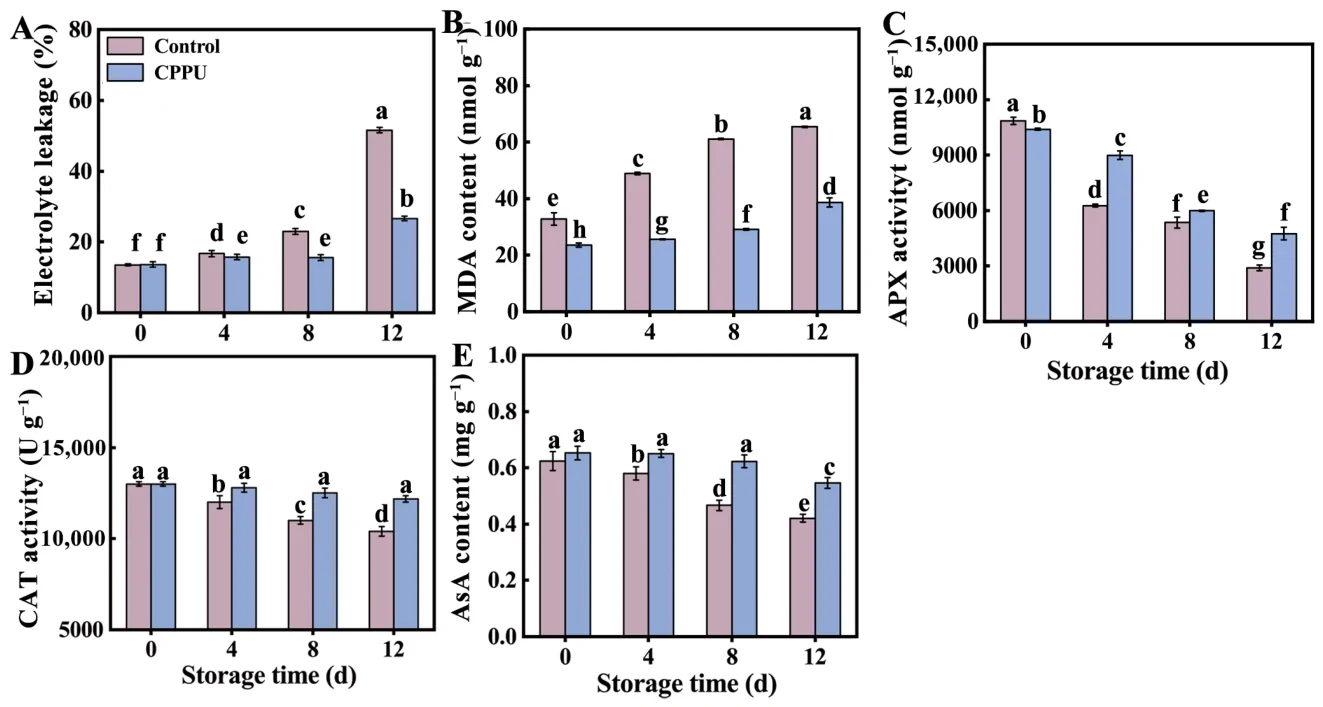
Changes in malondialdehyde (MDA) content, ascorbate peroxidase (APX) activity, and pericarp electrolyte leakage in preharvest CPPU-treated litchi fruit during 12 days of postharvest ambient storage. (**A**) Electrolyte leakage (%); (**B**) MDA content; (**C**) APX activity; (**D**) CAT activity; and (**E**) AsA content. Each value represents the means ± SE of three replicates (SE). Different lowercase letters label significant differences at *p* < 0.05, determined by two-way ANOVA, followed by Tukey’s multiple comparison test for all combinations of treatments (control and CPPU) and storage time points (0, 4, 8, and 12 d).

**Figure 6:**
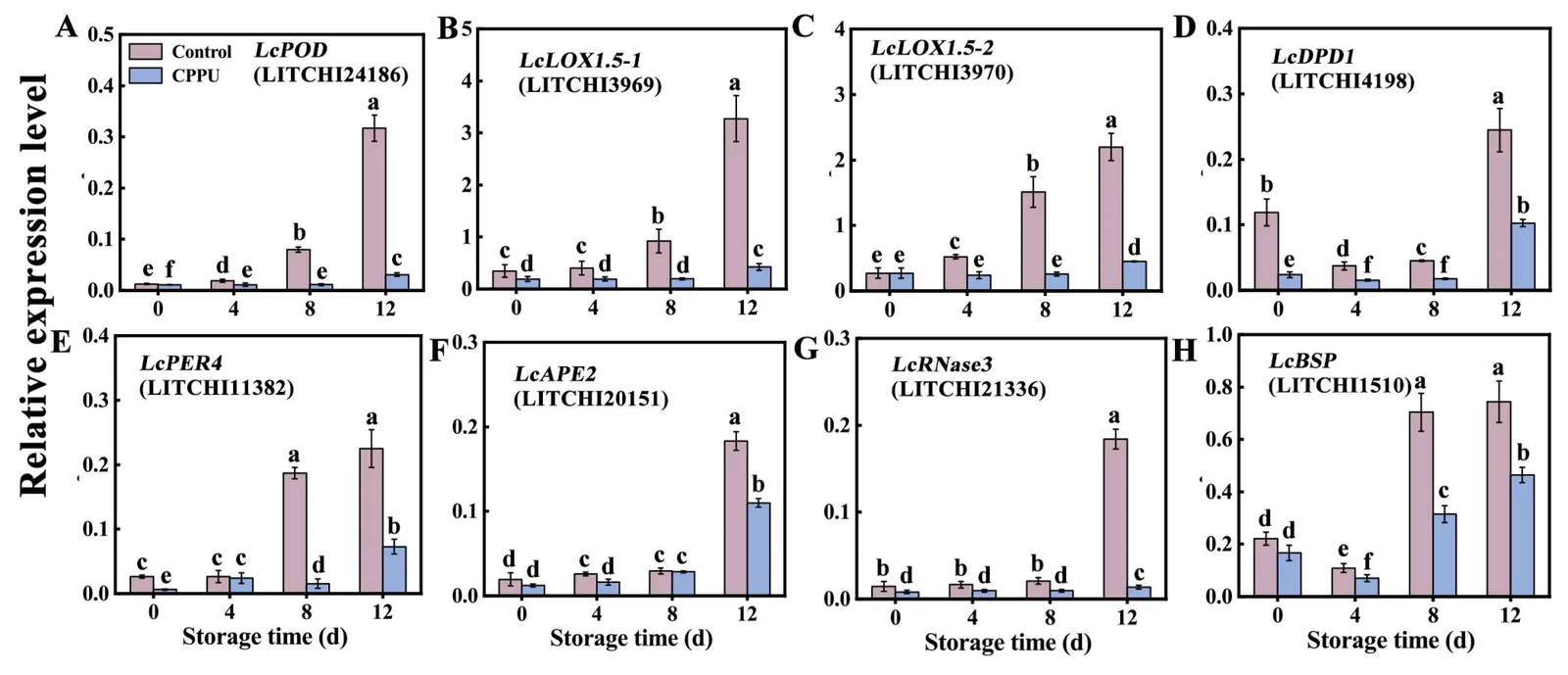
Gene expression of pericarp senescence-related genes in litchi during storage. (**A**) *LcPOD*; (**B**) *LcLOX1.5-1*; (**C**) *LcLOX1.5-2*; (**D**) *LcDPD1*; (**E**) *LcPER4*; (**F**) *LcAPE2*; (**G**) *LcRNase3*; (**H**) *LcBSP*.Each value represents the means ± SE of three replicates (SE). Different lowercase letters label significant differences at *p* < 0.05, determined by two-way ANOVA, followed by Tukey’s multiple comparison test for all combinations of treatments (control and CPPU) and storage time points (0, 4, 8, and 12 d).

**Figure 7:**
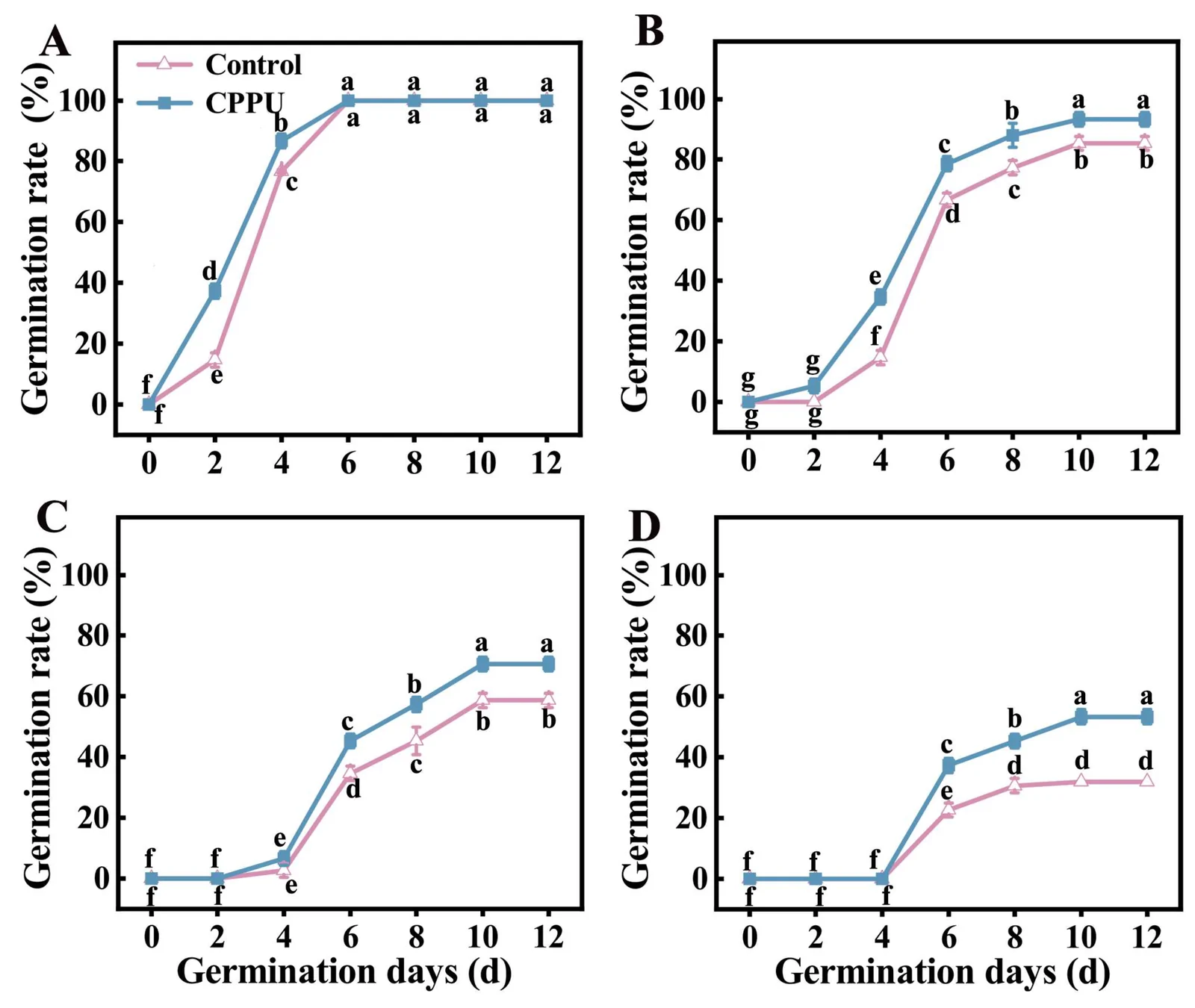
Cumulative germination rates of litchi seeds from the control and preharvest CPPU-treated fruit during the postharvest storage. (**A**–**D**) Germination curves of the seeds collected from the fruit after 0, 4, 8, and 12 d of postharvest storage, respectively. The *x*-axis represents incubation time during the germination assay, and the *y*-axis represents cumulative germination percentage (%). Each value represents the means ± SE of three replicates (SE). Different lowercase letters label significant differences at *p* < 0.05, determined by two-way ANOVA, followed by Tukey’s multiple comparison test for all combinations of treatments (control and CPPU) and germination time points (0, 2, 4, 6, 8, 10, and 12 d).

## Data Availability

The original contributions presented in this study are included in the article/Supplementary Materials. Further inquiries can be directed to the corresponding authors.

## Supplementary Materials

The following supporting information can be downloaded at https://www.mdpi.com/article/10.3390/foods15193458/s1, Table S1: The gene-specific primers used for RT-qPCR; Table S2: Content of phytohormone profiling the relative accumulation of auxin, gibberellin (GA), abscisic acid (ABA), and cytokinin-related metabolites in the pericarp and seed of the control and CPPU-treated fruit.
